# Anti-Cancer Stem-like Cell Compounds in Clinical Development – An Overview and Critical Appraisal

**DOI:** 10.3389/fonc.2016.00115

**Published:** 2016-05-10

**Authors:** Fabrizio Marcucci, Cristiano Rumio, François Lefoulon

**Affiliations:** ^1^Department of Pharmacological and Biomolecular Sciences, University of Milan, Milan, Italy; ^2^Technologie Servier, Department of Synthesis, Orléans, France

**Keywords:** cancer stem-like cells, epithelial–mesenchymal transition, therapy, biomarkers, classification, clinical development

## Abstract

Cancer stem-like cells (CSC) represent a subpopulation of tumor cells with elevated tumor-initiating potential. Upon differentiation, they replenish the bulk of the tumor cell population. Enhanced tumor-forming capacity, resistance to antitumor drugs, and metastasis-forming potential are the hallmark traits of CSCs. Given these properties, it is not surprising that CSCs have become a therapeutic target of prime interest in drug discovery. In fact, over the last few years, an enormous number of articles describing compounds endowed with anti-CSC activities have been published. In the meanwhile, several of these compounds and also approaches that are not based on the use of pharmacologically active compounds (e.g., vaccination, radiotherapy) have progressed into clinical studies. This article gives an overview of these compounds, proposes a tentative classification, and describes their biological properties and their developmental stage. Eventually, we discuss the optimal clinical setting for these compounds, the need for biomarkers allowing patient selection, the redundancy of CSC signaling pathways and the utility of employing combinations of anti-CSC compounds and the therapeutic limitations posed by the plasticity of CSCs.

## Cancer Stem-Like Cells – Origin, Functional Properties, and Markers

Cancer stem-like cells (CSCs) represent a subpopulation of tumor cells. They are highly active in generating new tumors upon implantation in laboratory animals, while most tumor cells have low tumor-forming ability (1). CSCs are also resistant to conventional chemotherapeutics, radiation, and targeted therapies (2–4), and have enhanced metastasis-forming potential (5). Drug resistance allows CSCs to survive current therapies and to be ultimately responsible for relapse (4).

The first demonstration of the existence of CSCs was brought in acute myeloid leukemia (AML) (6). This initial observation was followed in ensuing year by similar observations in solid tumors (7–11).

The CSC population may not necessarily represent the original tumor-initiating cell (TIC) (12) and there is plasticity in the general tumor such that cells can loose and reacquire a CSC-like phenotype. Overall, it appears likely that, at any given time, CSCs are the result of the convergent action of two main events. The first is the genetic (mutations, rearrangements, and/or deregulation of genes) and the epigenetic (microRNAs, alterations of the methylation profile of genes, or gene promoters) instability of tumor cells and the products that are expressed, e.g., oncogenes (13, 14), or repressed as a consequence of this instability (15). The second event is represented by stimuli from the tumor microenvironment (TME). These stimuli are similar to those that promote the epithelial–mesenchymal transition (EMT) of tumor cells (16). Induction and maintenance of CSCs in response to these stimuli, however, is not a direct effect on tumor cells but, rather, the result of a cross-talk between different cell types within the TME that is largely effected by extracellular mediators released in response to the stimuli (17). Of note, genetic instability of tumor cells can also lead to overexpression of extracellular mediators (18, 19), which may add to those released in response to stimuli from the TME. The contribution of these two main events may differ quantitatively and qualitatively in different tumors and, over time, even within the same tumor, and this variability may underlie the plasticity and heterogeneity of CSCs. In fact, tumors may have a small number of CSCs, others a relatively large number, and still other tumors may even lack detectable CSCs (20, 21). Even within individual tumors, CSCs may express different, only partially overlapping phenotypes (22).

Given the multiplicity of genetic and environmental stimuli that are at the origin of CSCs, it is equally not surprising that a large number of signaling pathways have been reported being involved in the induction and maintenance of CSCs. Since CSCs and normal stem cells share a number of traits (4), it is logical that the role of signaling pathways involved in the physiology of normal stem cells, such as WNT, Notch, and Hedgehog (Hh), has been investigated with particular attention (4, 23).

## The Relationship Between EMT and CSCs

**Figure 1:**
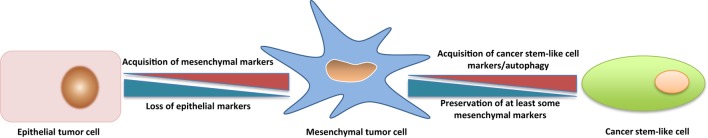
**Transition from a Tumor Cell with a Predominantly Epithelial Phenotype to a Tumor Cell with a Predominantly Mesenchymal Phenotype and to a Cancer Stem-Like Cell (CSC)**. The transition from a predominantly epithelial to a predominantly mesenchymal phenotype is better understood in molecular and phenotypic terms, but most available data suggest that a tumor cell with a predominantly mesenchymal phenotype must undergo several changes in order to acquire properties of a CSC, including autophagy. The heterogeneity of CSC populations suggests also the possibility of different transition states between a predominantly mesenchymal tumor cell and a CSC, in analogy with what has been observed for the transition between predominantly epithelial and mesenchymal tumor cells. See text for discussion and references to this point.

CSCs can derive from bulk tumor cells that undergo an EMT, i.e., the conversion of tumor cells with an epithelial phenotype into cells with a mesenchymal phenotype (24, 25). EMT is critical for embryonic development and involves changes that lead to loss of cell–cell adhesion and cell polarity, with acquisition of migratory and invasive properties (26). In adults, EMT occurs during wound healing, tissue regeneration, organ fibrosis, and tumor progression (27). Tumor cells undergoing EMT are characterized by increased motility and invasiveness, resistance to antitumor drugs, and acquire tumor-initiating potential (28, 29). Reversal of EMT is accompanied by downregulation of CSC-associated traits (30). The question that obviously arises is whether tumor cells that have undergone an EMT are CSCs, i.e., if the two terms are interchangeable. The question is not of mere academic interest because, in case of coincidence, therapeutic approaches aimed at targeting CSCs would be identical to those addressing EMT (31). Overall, there is considerable evidence for the two cell types not being coincident. Thus, it has been proposed that cells that have undergone an EMT acquire a CSC phenotype by engaging additional programs, such as the WNT and Hippo pathways (32). Furthermore, under certain circumstances, EMT and stemness can be uncoupled (33). Similarly, Thiery and coworkers have recently proposed that it is not solely the acquisition of EMT but the EMT stem cell-like phenotype that engenders drug resistance (34). In accordance with this view, it has been observed that the anti-CSC compound salinomycin (see below for more details on this compound) can cause cell death and decrease stem cell properties despite activation of EMT (35). Eventually, in many instances CSCs have been reported to be in a quiescent, autophagic state, that is the greatly different from mesenchymal-type tumor cells with enhanced invasive and migratory potential (36, 37). Blockade of autophagy has even been reported to reduce CSC activity (38). Overall, it seems that CSCs represent a further developmental stage that ensues after tumor cells have undergone EMT (Figure 1), and that autophagy may be a specific trait of such CSCs. CSCs, however, may not loose mesenchymal traits, as has been shown for circulating tumor cells (39).

## Anti-CSC Compounds in Clinical Development

### Search Criteria

A large number of compounds that have already received regulatory approval or are in clinical development have been tested for their anti-CSC activity. The goal of this article, however, is to address those compounds that are in clinical development for their anti-CSC activity. In order to identify these compounds, a search was performed in the ClinicalTrials.gov website using the key word CSC. Of the almost 3,500 clinical trials that were recovered, however, only a minute fraction were actually dealing with CSCs, while the vast majority were on stem cells used for transplantation. In the following, we realized that the few studies that were directly dealing with CSCs were only a fraction of those investigating anti-CSC compounds. Some other studies were recovered using the key word TIC. It became clear, however, that many studies that were investigating anti-CSC compounds did not use the terms CSC or TIC and, therefore, could not be recovered. For this reason, we expanded the study, including pharmaceutical companies known to be involved in anti-CSC drug discovery. Eventually, we included also antitumor compounds that are not or not yet being developed as anti-CSC compounds, but whose anti-CSC activities have been confirmed in numerous preclinical studies. On the other hand, we excluded compounds that have already received regulatory approval for antitumor indications unrelated to CSCs, but which have recently been found to possess some selectivity for CSCs [e.g., Ref. (40)]. Admittedly, this approach is somewhat arbitrary but it has allowed to keep the number of anti-CSC compounds to be discussed in a manageable size while, nevertheless, addressing all main classes of anti-CSC compounds with one or more compounds being discussed for each class.

### Anti-CSC Compounds Targeting Extracellular Mediators or Cell Surface Molecules

In this section, we discuss compounds that target molecules that are extracellular mediators or are expressed on the surface of CSCs. Extracellular mediators and cell surface molecules that are part of complex signaling pathways involved in CSC biology and can be targeted at different levels of these pathways are discussed in later sections.

A large number of cell surface molecules that are expressed on CSCs of tumors or tumor subtypes of different tissue origin have been identified. CD44, CD47, CD33, CD133, CXC chemokine receptor (CXCR) 4, and CD26 are just some of these markers (41). Most of them are not CSC specific and in some cases may even be ubiquitously expressed, including blood cells (e.g., CD44, CD47) (42). Targeting these markers for therapeutic purposes may incur into severe side effects. Some CSC markers, however, have a more restricted expression and/or are overexpressed on CSCs, making them good potential targets for anti-CSC compounds. Recent approaches, such as the construction of bispecific antibodies, may offer, for the future, the opportunity to target also CSC markers that are broadly expressed (43).

#### Inhibitors of Clusterin

Clusterin is a stress-activated and apoptosis-associated molecular chaperone that protects cells from various stressors and is overexpressed in many human cancers (44). Antibodies targeting secreted clusterin inhibit transforming growth factor (TGF)-β-induced EMT in several tumor cell lines, without affecting the proliferation, and reduce lung metastasis in breast cancer and hepatocellular carcinoma (HCC) models (45, 46). Although no formal proof has been brought that anti-clusterin antibodies inhibit also CSCs, a humanized anti-clusterin mAb (AB-16B5) is being investigated in patients with solid tumors for clinical efficacy, and effects on EMT and CSC biomarkers (Table 1).

**Table 1:** **Anti-CSC compounds targeting cell surface molecules**.

Anti-CSC compound	ClinicalTrials.gov Identifier (phase for the indicated trials): patient population, CSC-related outcomes	Reference
Anti-clusterin mAb (AB-16B5) 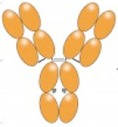	NCT02412462 (phase I): advanced solid tumors. Among outcomes: EMT and CSC biomarkers in circulating tumor cells and tumor biopsies	(45)
CXCR1 antagonist (reparixin) 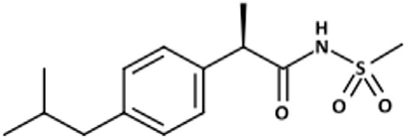	NCT02001974 (phase Ib, completed): HER2-negative metastatic breast cancer. Among outcomes: expression of ALDH1 and CD44 on tumor biopsies	(47)
Anti-ROR-1 mAb (cirmtuzumab/UC-961) 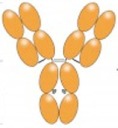	NCT02222688 (phase I): relapsed or refractory CLL ineligible for chemotherapy	(48)

*ALDH, aldehyde dehydrogenase; CLL, chronic lymphocytic leukemia; CRC, colorectal cancer; CSC, cancer stem-like cell; CXCR, CXC chemokine receptor; EMT, epithelial–mesenchymal transition; HER, human epidermal growth factor receptor; mAb, monoclonal antibody; ROR-1, receptor tyrosine kinase-like orphan receptor 1; RSPO3, R-spondin-3*.

#### Inhibitors of CXCR1

Gene expression profiling of tumor cell lines that had been enriched for aldehyde dehydrogenase (ALDH)-positive CSCs identified interleukin (IL)-8 and its receptor CXCR1 as constitutive of the CSC profile (49). IL-8 was also found to increase the number of ALDH-positive tumor cells and mammosphere formation *in vitro*, an assay that reflects the tumor-initiating potential of cancer cells. Later, the same group showed that CXCR1 blockade using either a CXCR1-specific blocking antibody or reparixin, a methanesulfonamide CXCR1 inhibitor, depleted the CSC population in two human breast cancer cell lines *in vitro* (50) and targeted the CSC population in breast cancer xenografts, retarding tumor growth and reducing metastasis. Another work showed that this compound, in combination with paclitaxel, inhibited formation of brain metastases in a breast cancer model (47). This was likely the result of the combined effect of the two drugs, the pro-apoptotic action of paclitaxel and the cytostatic and anti-migratory effects of reparixin. Reparixin has been investigated in a clinical study in patients with human epidermal growth factor receptor (HER) 2-negative metastatic breast cancer (Table 1).

#### Inhibitors of Receptor Tyrosine Kinase-Like Orphan Receptor 1

Receptor tyrosine kinase-like orphan receptor 1 (ROR1) is a type I orphan receptor, tyrosine kinase-like cell surface protein that is expressed during embryogenesis and is found on tumor cells of many different types of cancer, but not on normal adult tissues (51). ROR1 is preferentially expressed by less well-differentiated tumors with EMT-related markers that have high potential for relapse and metastasis. Silencing ROR1 in breast cancer cell lines attenuated expression of EMT-associated genes and impaired their metastatic potential *in vivo* (51). A recent study has reported that ROR1 is associated with ovarian cancer CSCs (48). Cirmtuzumab/UC-961, a humanized IgG1 mAb, binds with high-affinity ROR1, and inhibits migration *in vitro* and engraftment in mice of patient-derived tumor cells that had been treated with the antibody (48). Cirmtuzumab is currently being investigated in patients with chronic lymphocytic leukemia who are ineligible for chemotherapy (Table 1).

### Anti-CSC Compounds That Act on Ligand–Receptor Pairs and Their Signaling Pathways

#### Inhibitors of the TGF-β/TGF-β Receptor Pathway

The TGF-β/TGF-β receptor pathway is one of the most frequently involved in EMT and CSC biology. A recent study showed that blocking TGF-β signaling with a TGF-β type I receptor kinase inhibitor, EW-7197, suppressed paclitaxel-induced EMT and CSC functions, such as formation of mammospheres and ALDH activity, reduced the ratio of CD44^+^/CD24^−^ CSCs, and CSC-associated transcription factors (52). Treatment with EW-7197 improved the efficacy of paclitaxel by decreasing the number of lung metastases and increasing survival time *in vivo*. The TGF-β pathway has also been shown to cross-talk with other signaling pathways involved in CSC biology, such as the Notch pathway (53). Thus, heightened Notch signaling in tumor cells magnified TGF-β-induced phosphorylation of signaling components and was required to sustain TGF-β-induced lung carcinoma cell growth. Conversely, Notch blockade reduced TGF-β signaling and limited lung carcinoma tumor progression. Overall, the TGF-β/TGF-β receptor signaling axis is involved in the CSC biology of several tumor types, such as breast cancer (54, 55), liver cancer (56), lung cancer (57), and head and neck cancer (53).

With regard to the clinical development of anti-TGF-β compounds, it must be considered that TGF-β is a multifunctional cytokine and its inhibition leads to effects that are likely unrelated to EMT or CSC inhibition, such as enhancement of adaptive antitumor immune responses or normalization of the tumor stroma (58, 59). Nevertheless, several inhibitors of this pathway are now in clinical development, both anti-ligand antibodies (60) and inhibitors of the TGF-β receptor tyrosine kinase (RTK). One of the most frequently investigated for its anti-CSC activity is the TGF-β type I RTK inhibitor galunisertib/LY2157299. In a preclinical study, this compound blocked paclitaxel-induced CSC expansion in triple-negative breast cancer (TNBC) cell lines and mouse xenografts where the chemotherapeutic drug paclitaxel increased autocrine TGF-β signaling and IL-8 expression and enriched for CSCs (61). Moreover, treatment with LY2157299 prevented reestablishment of tumors after paclitaxel treatment. The clinical development of this TGF-β inhibitor has been delayed owing to the detection of cardiovascular toxicity in preclinical studies (62, 63). This problem has now been bypassed through the application of judicious administration protocols (64). LY2157299 is now in several clinical trials although none of these trials explicitly refers to its anti-CSC activity (see Table 2).

**Table 2:** **Anti-CSC compounds that act on ligand–receptor pairs and their signaling pathways**.

Anti-CSC compound	ClinicalTrials.gov Identifier (phase for the indicated trials): patient population, CSC-related outcomes	Reference
TGF-βIR TKI: galunisertib/LY2157299 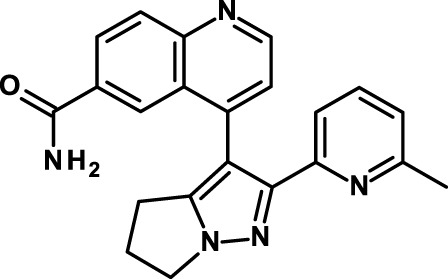	NCT02240433, NCT02178358, NCT01246986 (phases Ib–II): in advanced hepatocellular carcinoma, alone or in combination with sorafenib NCT02154646, NCT01373164 (phases Ib–II): with gemcitabine in unresectable pancreatic cancer NCT02452008 (phase II): with enzalutamide in metastatic prostate cancer NCT01220271, NCT01582269 (phases Ib–II): with radiochemotherapy or lomustine in malignant glioma NCT02538471 (phase II): with radiotherapy in metastatic breast cancer NCT02423343 (phase Ib/II): with anti-PD-1 (nivolumab) in different recurrent or refractory solid tumors	(61)
Hh-SMO inhibitor: vismodegib 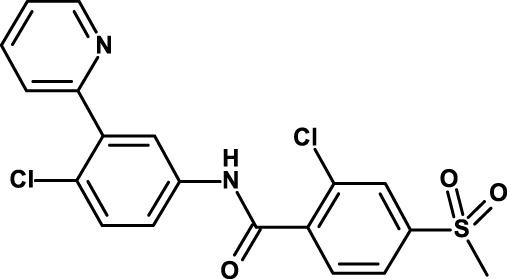	NCT01195415 (phase II, completed): with gemcitabine in advanced pancreatic cancer Among outcomes: percent decrease of CD44^+^/CD24^+^ cells from biopsy NCT01088815 (phase II): with gemcitabine and nab-paclitaxel in previously untreated metastatic pancreatic cancer. Among outcomes: changes in pancreatic CSCs	(65) (66) (67, 68)
Hh-SMO inhibitor: saridegib/IPI-926 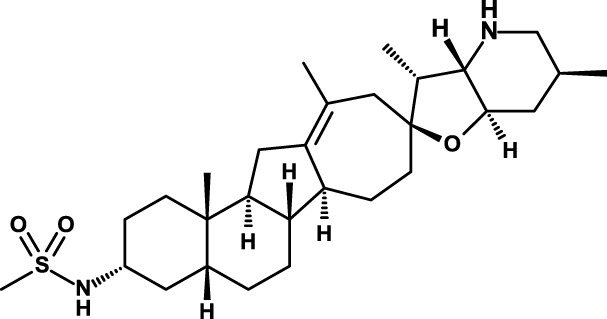	NCT01255800 (phase I): with cetuximab in recurrent head and neck cancer	(69) (70)
Anti-RSPO3 mAb: OMP-131R10 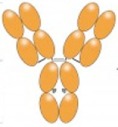	NCT02482441 (phase Ia/Ib): in combination with chemotherapy in previously treated metastatic CRC	(71)
Anti-Frizzled receptors mAB: vantictumab/OMP-18R5 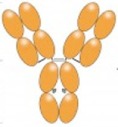	NCT01957007 (phase Ib): with docetaxel in patients with previously treated NSCLC NCT02005315 (phase Ib): with Nab-paclitaxel and gemcitabine in untreated stage IV pancreatic cancer NCT01973309 (phase Ib): with paclitaxel in locally recurrent or metastatic breast cancer	(72)
Frizzled 8 receptor-IgG1 Fc fusion: ipafricept/OMP-54F28 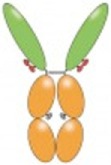	NCT02092363 (phase Ib): with paclitaxel and carboplatin in recurrent platinum-sensitive ovarian cancer	(73)
WNT, inhibitor of the interaction β-catenin-TCF: CWP232291 Undisclosed structure	NCT02426723 (phase Ia/Ib): alone or with lenalidomide and dexamethasone in relapsed or refractory multiple myeloma NCT01398462 (phase I): in various relapsed or refractory hematological malignancies	(74)
Inhibitor of the interaction β-catenin-TCF: PRI-724 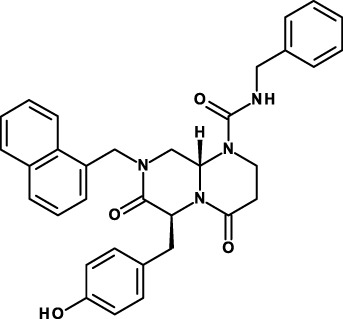	NCT01764477 (phase Ib): in advanced or metastatic pancreatic cancer eligible for second-line therapy NCT02413853 (phase II): with or without chemotherapy/anti-VEGF mAb as first line treatment for metastatic CRC NCT01606579 (phase I/II): in advanced myeloid malignancies	(75) (76)
Inhibitor of the interaction β-catenin–mucin-1: GO-203-2C Ac-(d)Arg-(d)Arg-(d)Arg-(d)Arg-(d)Arg-(d)Arg-(d)Arg-(d)Arg-(d)Arg-(d)Cys-(d)Gln-(d)Cys-(d)Arg-(d)Arg-(d)Lys-(d)Asn-NH_2_ disulfide	NCT02204085 (phase I/II): in relapsed or refractory AML. Among outcomes: assess whether GO-203-2c is effective in targeting mucin-1-C overexpressing AML progenitor cells, in decreasing engraftment potential of AML progenitor cells	(77) (78)
Notch, anti-DLL4 mAb: demcizumab/OMP-21M18 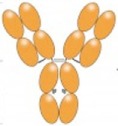	NCT01189929, NCT02289898 (phases Ib–II): with gemcitabine ± Abraxane^®^ in pancreatic cancer. Among outcomes: assessment of exploratory biomarkers NCT01952249 (phase Ib/II): with paclitaxel in platinum-resistant ovarian, peritoneal, or fallopian tube cancer NCT01189968, NCT02259582 (phase Ib-II): with carboplatin and pemetrexed in non-squamous NSCLC. Among outcomes: assessment of biomarkers NCT01189942 (phase Ib): with chemotherapy as first- or second-line treatment metastatic CRC. Among outcomes: assessment of biomarkers	(79)
Notch, bispecific anti-DLL4 x anti-VEGF mAb: OMP-305B83 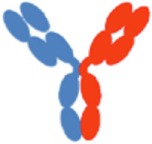	NCT02298387 (phase I): dose escalation and expansion study in solid tumors	
Anti-Notch 2/3 mAb: tarextumab/OMP-59R5 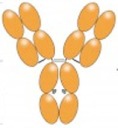	NCT01859741 (phase Ib/II): with etoposide and platinum therapy in untreated extensive stage SCLC NCT01647828 (phase Ib/II): with nab-paclitaxel and gemcitabine in untreated stage IV pancreatic cancer	(30)
Anti-Notch 1 mAb: brontictuzumab/OMP-52M51 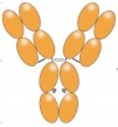	NCT01778439 (phase I): dose escalation study in solid tumors NCT01703572 (phase I): dose escalation study in lymphoid malignancies	
Anti-DLL3 ADC: rovalpituzumab tesirine/SC16LD6.5 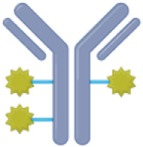	NCT01901653 (phase I/II): dose escalation study of safety, pharmacokinetics, and preliminary efficacy in recurrent SCLC	(80)
Anti-Ephrin A4 ADC: PF-06647263 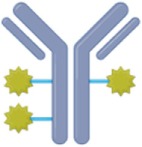	NCT02538471 (phase I): dose escalation, safety, and pharmacokinetics study in advanced solid tumors	(81)

*ADC, antibody-drug conjugate; AML, acute myeloid leukemia; CRC, colorectal cancer; CSC, cancer stem-like cell; CXCR, CXC chemokine receptor; DLL, Delta-like ligand; EMT, epithelial–mesenchymal transition; Fc, fraction crystallizable; Hh, Hedgehog; IgG, immunoglobulin G; mAb, monoclonal antibody; NSCLC, non-small cell lung cancer; PD, programed cell death; SMO, smoothened; TCF, T-cell factor; TGF-βIR, transforming growth factor-βI receptor; TKI, tyrosine kinase inhibitor; VEGF, vascular endothelial growth factor*.

#### Inhibitors of the Hedgehog Pathway

This signal transduction pathway, as well the WNT and Notch pathways are crucial for embryonic development. Therefore, compounds targeting these pathways may have negative consequences on embryonic patterning and child development. Furthermore, these pathways are utilized in the generation of stem cells and in regenerative processes, and compounds acting on these pathways may have untoward effects on these processes (82). In spite of these concerns, an inhibitor of this pathway, vismodegib, has received regulatory approval (83).

In adult tissues, Hh signaling is relatively quiescent except in tissue maintenance and repair. Aberrant activation of Hh signaling is implicated in multiple aspects of tumorigenesis, including maintenance of the CSC phenotype (84). Canonical Hh signaling is activated when a Hh ligand (Sonic Hh, Indian Hh, and Desert Hh) binds the transmembrane receptor Patched (PTCH) to relieve PTCH-mediated inhibition of the G-protein-coupled receptor-like protein Smoothened (SMO). SMO then drives a signaling cascade that results in nuclear translocation and activation of the glioma-associated oncogene transcription factors (GLI). GLI activate transcription of genes regulating self-renewal, cell fate, survival, angiogenesis, EMT, and cell invasion.

Both ligand-dependent and ligand-independent mechanisms (instabilities of genes encoding individual components that lead to constitutive activation of the pathway) result in aberrant Hh pathway activation in cancer. Hh signaling associates with CSC biology in several types of hematological malignancies and solid tumors, such as pancreatic cancer (85), prostate cancer (86), glioblastoma (87), lung cancer (88), breast cancer (89), colon cancer (90), chronic myeloid leukemia (91), and multiple myeloma (92). Preferential activation of Hh signaling in CSCs compared with bulk tumor cells has been reported (88–90, 92) as well as upregulation of CSC markers (65, 89, 93). Several inhibitors, mostly small-molecule inhibitors, of the Hh pathway are in clinical development for their antitumor effects. One of these, vismodegib, has already received regulatory approval. Some of these inhibitors, including vismodegib, are also being tested clinically for their anti-CSC activity.

The first small molecule that was found to inhibit the Hh pathway is cyclopamine, a naturally occurring compound that belongs to the group of *jerveratum* alkaloids (94). Pharmaceutical companies have set out to develop cyclopamine derivatives with improved pharmacologic properties or new molecules showing improved binding to SMO, so far the main target for Hh pathway inhibitors. Vismodegib has been approved for the treatment of advanced basal cell carcinoma, where it induces a high percentage of response rates (95), but inevitably incurs into acquired resistance (96). Vismodegib has demonstrated good efficacy also in medulloblastoma (97), but only limited activity in other tumor types. This lack of activity may be due to many factors, but the possibility of a tumor type-dependent redundancy of signaling pathways appears a likely possibility.

In preclinical studies, vismodegib inhibited cell viability and induced apoptosis in three pancreatic cancer cell lines and pancreatic CSCs (66). Suppression of both GLI1 plus GLI2 mimicked the changes in cell viability, spheroid formation, apoptosis, and gene expression observed in vismodegib-treated pancreatic CSCs. In another study, vismodegib decreased spheroid and colony formation of gastric cancer cell lines with upregulated CD44 and Hh pathway proteins (65). CD44-positive cells were more resistant to chemotherapeutics, showed enhanced migration, invasion, and anchorage-independent growth, and these properties were reversed by vismodegib. Vismodegib is being investigated in two phase II clinical studies for its anti-CSC activity. In both, vismodegib is studied in advanced pancreatic cancer in combination with chemotherapeutics (Table 2). Results of one of these studies have been published (67). Treatment for 3 weeks led to down-modulation of GL1 and PTCH1 and decreased fibrosis, but no significant changes in CSCs were observed, and combined treatment with vismodegib and gemcitabine was not superior to gemcitabine alone.

Saridegib/IPI-926 is another SMO inhibitor that is in clinical investigation (69). It is being studied in combination with the anti-epidermal growth factor receptor (EGFR) mAb cetuximab in recurrent head and neck cancer patients (Table 2). Preliminary results of this study have now been published (70). Among eight evaluable patients, the best responses were one partial response, four stable diseases, and three disease progressions. Decreases in tumor size were seen in both cetuximab-naïve patients. Toxicities were as expected. Tumor shrinkage and progression-free survival were associated with downregulation of intra-tumoral Hh pathway gene expression during therapy.

#### Inhibitors of the WNT Pathway

WNT proteins are a large family of secreted molecules that play a critical role in the development of various organisms (98). In the absence of extracellular WNT molecules, a destruction complex, including the proteins adenomatous polyposis coli (APC), glycogen synthase kinase 3β (GSK3β) and AXIN, phosphorylates β-catenin, targeting it for ubiquitylation and degradation. The binding of WNTs to Frizzled receptors and the co-receptors low-density lipoprotein receptor-related protein 5 (LRP5) and LRP6, transmits a signal through Dishevelled, which results in inhibition of the destruction complex and nuclear entry of β-catenin. In the nucleus, β-catenin acts as a bridge between members of the T cell factor (TCF) family of transcription factors and the basal transcriptional apparatus via co-activators [CREB-binding protein (CBP), E1A-associated protein p300, the co-activator Pygopus, B cell lymphoma 9, etc]. Aberrant activation of the WNT/β-catenin signaling pathway has recently been implicated in several types of human cancers (82), and shown to play a critical role in CSC biology (99, 100). Moreover, aberrant activation of the transcriptional activity of β-catenin, independently of upstream WNT signaling, has been associated with breast CSC amplification and tumorigenesis (101).

Inhibitors of the WNT pathway may act at different levels of the signaling chain. One approach is to act on ligands of this pathway. R-spondins (RSPOs) are secreted proteins that potentiate canonical WNT signaling (102). Translocations of RSPO genes are recurrent in a subset of colorectal tumors. PTPRK–RSPO3 is one of the fusions that can originate from these translocations. It has recently been shown that targeting RSPO3 in PTPRK–RSPO3-fusion-positive human tumor xenografts inhibits tumor growth and promotes differentiation (71). Genes expressed in the stem-cell compartment of the intestine were among those most sensitive to anti-RSPO3 treatment. A clinical study with the anti-RSPO3 mAb OMP-131R10 is ongoing in advanced solid tumors and in metastatic colorectal cancer (Table 1).

Another approach has been to raise inhibitory mAbs against Frizzled receptors. This is a challenging undertaking since there are 19 human WNTs and 10 Frizzled receptors. Nevertheless, a mAb, vantictumab/OMP-18R5, which binds to five distinct Frizzled receptors through a conserved epitope, has been obtained (72). In xenograft studies with minimally passaged human tumors, this antibody inhibited the growth of a range of tumor types, reduced tumor cell proliferation, and CSC frequency. Strong synergy was observed with several chemotherapeutics. Vantictumab is now being investigated in several phase I clinical trials in combination with chemotherapeutics in patients with advanced solid tumors (Table 2).

The fusion protein ipafricept/OMP-54F28 encompasses a portion of the Frizzled 8 receptor fused to the Fc portion of IgG1. This protein competes with the native Frizzled 8 receptor for its ligands and antagonizes WNT signaling. In preclinical models, OMP-54F28 reduced tumor growth and decreased CSC frequency as a single agent and in combination with chemotherapeutics (73). A phase Ia clinical study is nearing completion with this compound in advanced solid tumors and phase Ib studies have been started in combination with chemotherapeutics in ovarian, pancreatic, and hepatocellular cancers (Table 2).

Organic molecules have been synthesized that antagonize the binding of β-catenin to the TCF protein or to transcriptional co-activators. These molecules cause downregulation of β-catenin-responsive genes. CWP232228 inhibits the interaction of β-catenin with TCF (74), and this was shown to inhibit the growth of both breast CSCs and bulk tumor cells, but breast CSCs exhibited greater sensitivity. CWP232228 treatment blocked also secondary xenograft tumor development and inhibited metastasis formation. CWP232228 is not being investigated in clinical studies, but an undisclosed, but probably closely related molecule (CWP232291) is now in phase I clinical studies for the treatment of hematological malignancies (Table 2).

PRI-724 is a small molecule that inhibits the interaction between β-catenin and CBP. PRI-724 sensitized ovarian cancer cells to cisplatin and decreased tumor sphere formation (75). Importantly, CBP/β-catenin antagonists appear to have the ability to safely eliminate CSCs by taking advantage of an intrinsic differential preference in the way somatic stem cells and CSCs divide (76). PRI-724 is currently in phase I/II clinical trials for the treatment of solid tumors and hematological malignancies (Table 2).

Still another approach to interfere with this pathway is to inhibit the interaction between mucin 1 and β-catenin. Mucin 1 is a membrane-bound glycoprotein expressed by most glandular and ductal epithelial cells, and is overexpressed in many diverse human carcinomas. The mucin 1–β-catenin interaction is stabilized by extracellular matrix protein 1 and leads to the upregulation of EMT- and CSC-related genes (77, 78). The mucin 1 inhibitor GO-203-2C is a highly cationic peptide with a polyarginine tail at the N-terminus. It interacts with the mucin 1 C-terminus on the cell surface and inhibits cell–cell interactions and signaling. It is currently in a phase I/II trial in patients with relapsed or refractory AML (Table 2).

Several other small-molecule WNT/β-catenin signaling inhibitors have been developed and are in development [listed in Ref. (74)], but so far no direct evidence has been brought that these molecules inhibit CSCs, although this appears a likely possibility.

#### Inhibitors of the Notch Pathway

The Notch pathway regulates cell fate specification, tissue patterning, and cellular survival. In mammals, the Notch signaling system consists of five membrane-bound ligands, DLL1, 3, 4, and Jagged 1, 2, and four single-pass transmembrane receptors, Notch1–4. Ligand binding to Notch triggers the proteolytic activation of the receptor and translocation of its intracellular domain to the nucleus, where it interacts with the CSL transcription factor to regulate the expression of target genes (103). Notch signaling is involved in normal development and in most organs, including the hematopoietic system and the vasculature (103). Alterations in the Notch signaling pathway stimulate proliferation, restrict differentiation, promote cellular survival, and are associated with oncogenesis in several malignancies. Notch plays a critical role in CSC biology (86). Of the Notch ligands, DLL4 is commonly expressed in solid tumors and associated with chemoresistance. DLL4 binds all four Notch receptors, but the interaction with Notch 1 is preferred (103).

Several approaches are being pursued for inhibition of Notch signaling. A first has been to inhibit Notch receptor cleavage by γ-secretase inhibitors (104). The therapeutic utility of these compounds, however, is limited due to intestinal toxicity resulting from pan-Notch inhibition (105). One γ-secretase inhibitor (RO4929097) was being studied in clinical trials for its antitumor and anti-CSC activity in breast cancer, but its development has now been discontinued.

A second approach has been to generate mAbs that inhibit Notch signaling. A humanized IgG2 anti-DLL4 mAb, demcizumab/OMP-21M18, is now in clinical investigation as anti-CSC compound (79) (Table 2). In human tumor models, both alone or in combination with chemotherapy, it reduced tumor growth, regrowth, and number of CSCs. A phase I clinical trial has allowed defining a recommended phase 2 dose. Moreover, 16 of 25 (64%) evaluable patients at one dose (10 mg/kg) had evidence of disease stabilization or response (79). Results suggested also that the antitumor effects of demcizumab might be the result of a combination of anti-CSC and anti-angiogenic effects, an observation consistent with the role of Notch signaling in normal angiogenesis (106) and tumor angiogenesis (107).

Given the role of Notch signaling in tumor angiogenesis, it is not surprising that other anti-DLL4 mAbs are in clinical investigation as inhibitors of tumor angiogenesis (e.g., enoticumab) (108). In fact, one compound in clinical development has been designed to optimally exploit both anti-angiogenic and anti-CSC activities. This is OMP-305B83, a bispecific anti-DLL4 and anti-vascular endothelial growth factor (VEGF) mAb that is currently in phase I clinical studies (Table 2).

In addition to inhibit the activity of Notch ligands, another possibility is to inhibit this pathway with anti-receptor (anti-Notch) antibodies. In order to avoid pitfalls deriving from pan-Notch inhibition, a novel mAb, tarextumab/OMP-59R5, has been generated that blocks the function of Notch 2 and Notch 3 (30). OMP-59R5 reduced CSC frequency in combination with chemotherapeutic agents in various cancer models, and the triple combination of anti-Notch2/3 with gemcitabine plus nab-paclitaxel produced striking tumor regression in pancreatic cancer xenografts. OMP-59R5 is now in phase I clinical trials (Table 2). Still another mAb that is in clinical trials binds only the Notch1 receptor (brontictuzumab/OMP-52M51). It is employed only for patients that demonstrate tumor overexpression of the activated form of Notch1. For this purpose, patients’ tumors are prescreened by immunohistochemistry to determine eligibility (Table 2).

A special role in the Notch pathway is played by DLL3. DLL3 predominantly localizes to the Golgi apparatus and is unable to activate Notch signaling (109). In the course of normal development, DLL3 inhibits Notch pathway activation by interacting with Notch and DLL1 and redirecting or retaining them to the late endosomal/lysosomal compartments or in the Golgi, respectively, thereby preventing their localization to the cell surface (110). DLL3 is overexpressed and relocalizes to the surface of small cell lung cancer (SCLC) and large cell neuroendocrine carcinoma cells (80). A DLL3-targeted antibody-drug conjugate (ADC), rovalpituzumab tesirine/SC16LD6.5, encompassing a humanized anti-DLL3 mAb conjugated to a DNA-damaging pyrrolobenzodiazepine dimer was synthesized and found to induce durable tumor regression *in vivo* across multiple patient-derived xenograft models. Lack of tumor recurrence resulted from effective targeting of DLL3-expressing CSCs. *In vivo* efficacy correlated with DLL3 expression, and responses were observed in xenograft models independently of their sensitivity to standard-of-care chemotherapy regimens. This ADC is now being investigated in a phase I/II clinical trial in patients with SCLC (Table 2).

#### Inhibitors of the Ephrin/Ephrin Receptor Pathway

Ephrin receptors comprise the largest family of RTKs in the human genome and modulate signaling pathways that impact cell fate decisions during embryogenesis and adult tissue homeostasis (111). Numerous therapeutics targeting this pathway are being tested in clinical trials; the majority are tyrosine kinase inhibitors. Vast functional redundancy within the Ephrin/Ephrin receptor pathway likely compromises the effect of blocking specific Ephrin ligands, while pan-Ephrin inhibition is toxic (111).

Gene expression analysis of breast and ovarian CSCs identified Ephrin A4 as a potential therapeutic target (81). An ADC targeting Ephrin A4 has been generated in order to induce cell death upon internalization, thereby avoiding redundancy of this highly diverse ligand–receptor family, and delaying the insurgence of resistance. This conjugate, labeled PF-06647263, encompasses a humanized anti-Ephrin A4 mAb conjugated to the DNA-damaging agent calicheamicin. It achieved sustained tumor regressions in both TNBC and ovarian cancer xenografts *in vivo* (81). Non-claudin low TNBC tumors exhibited higher expression and more robust responses than other breast cancer subtypes. PF-06647263 is currently being evaluated in a phase I clinical trial.

### Anti-CSC Compounds That Inhibit Post-Receptor Signaling Pathways

#### Inhibitors of SRC and Focal Adhesion Kinase

SRC and focal adhesion kinase (FAK) are two interacting membrane-proximal tyrosine kinases. Both can be activated by adhesion receptors, RTKs, and cytokine receptors. Increased SRC and FAK phosphorylation and activity are upregulated in many cancers and have been implicated in several aspects of cancer progression. Both of these tyrosine kinases have been implicated in CSC biology. SRC has been reported being involved in CSC biology of, for example, hepatic cancer (112), pancreatic cancer (113), and breast cancer (114); FAK in breast cancer (115) and squamous cell carcinoma (116).

Several SRC and FAK inhibitors are in clinical studies. Some of these studies are centered on their anti-CSC activity. Dasatinib is a non-specific SRC inhibitor that inhibits also the Bcr/Abl tyrosine kinase. It has been approved for the treatment of chronic myeloid leukemia and Philadelphia chromosome-positive acute lymphoblastic leukemia. Dasatinib was shown in a clinical study to induce a long-standing decrease of leukemic stem cells in patients with CML (117). It is unclear, however, whether this effect is the result of Bcr/Abl inhibition in this patient population or whether the decrease is the result of inhibition of both tyrosine kinases. Dasatinib is being studied in a phase II clinical trial for the depletion of CSCs in CML patients (Table 3).

**Table 3:** **Anti-CSC compounds that inhibit post-receptor signaling pathways**.

Anti-CSC compound	ClinicalTrials.gov Identifier (phase for the indicated trials): patient population, CSC-related outcomes	Reference
SRC inhibitor dasatinib 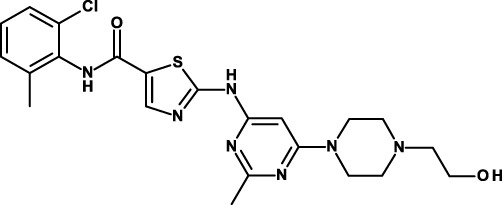	NCT00852566 (phase II): comparing depletion of CSCs with dasatinib vs. imatinib in newly diagnosed chronic myeloid leukemia	(117)
FAK inhibitor: defactinib/VS-6063/PF-04554878 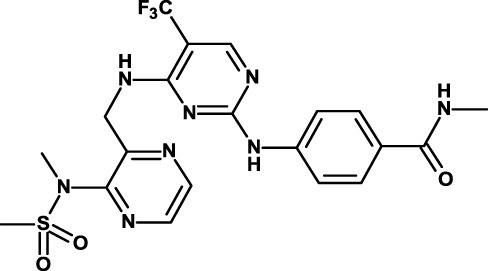	NCT01951690 (phase II): in KRAS mutant NSCLC. Phase II. Among outcomes: association between pharmacodynamic biomarkers and clinical outcome NCT01943292 (phase I): dose escalation to evaluate safety and pharmacokinetics in non-hematologic malignancies NCT01778803 (phase I/Ib): with paclitaxel in advanced ovarian cancer. Among outcomes: association between pharmacodynamic biomarkers and clinical outcome NCT02004028 (phase II): in surgical resectable malignant pleural mesothelioma. Among outcomes: biomarker responses in tumor tissue	(118)
	NCT00787033 (phase I): dose escalation to evaluate safety, pharmacokinetics, pharmacodynamics in advanced non-hematologic malignancies	(119)
FAK inhibitor: VS-4718 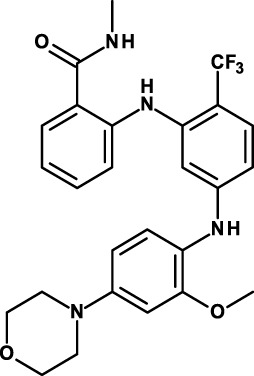	NCT01849744 (phase I): in metastatic non-hematologic malignancies. Among outcomes: correlation of biomarkers (phospho-FAK, CSCs) with response to VS-4718 therapy NCT02651727 (phase I): with Nab-paclitaxel and gemcitabine in advanced cancers	(120)
PI3K/mTOR dual inhibitor: VS-5584 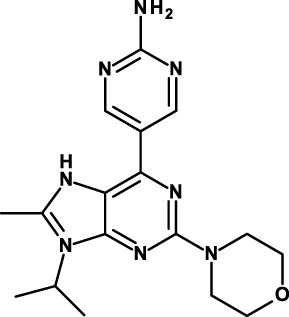	NCT01991938 (phase I): dose-escalation study in advanced non-hematologic malignancies or lymphoma. Among outcomes: correlation of tumor genetic alterations and/or biomarkers with response to therapy NCT02372227 (phase I): with FAK inhibitor VS-6063 in relapsed malignant mesothelioma	(121)
mTOR inhibitor temsirolimus 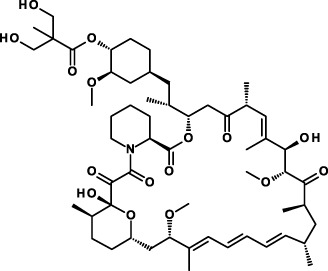	NCT00949325 (phase I/II): with liposomal doxorubicin in advanced soft tissue and bone sarcomas	(122)
MEK inhibitor: trametinib/GSK1120212 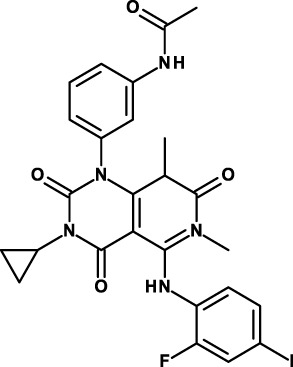	NCT01553851 (phase II): in surgically resectable oral cavity squamous cell cancer. Among outcomes: tumor-specific changes in putative CD44^+^ CSCs and intracellular phospho-extracellular signal-regulated kinase 1/2 staining after treatment	(123)
STAT3 inhibitor: Napabucasin/BBI608 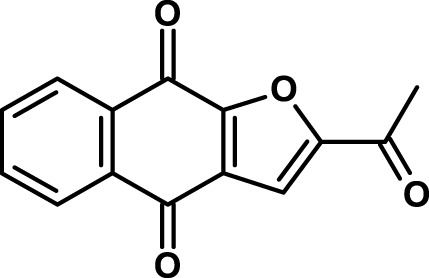	NCT02178956 (phase III): with paclitaxel vs. placebo + paclitaxel in gastric and gastro-esophageal junction cancer NCT01830621 (phase III): BBI608 and best supportive care vs. placebo and best supportive care in patients with pretreated advanced CRC NCT02315534 (phase Ib/II): with temozolomide in recurrent or progressed glioblastoma. Among outcomes: assessment of CSCs after tumor biopsy NCT02467361 (phase Ib/II): with immune checkpoint inhibitors to adult patients in advanced cancers. Among oucomes: CSC assays on biopsied patient tumor tissue NCT01325441 (phase Ib/II): with paclitaxel in advanced malignancies NCT02352558 (phase Ib): in advanced, refractory hematologic malignancies. Among outcomes: CSC assays on patient samples NCT02024607 (phase Ib): with standard chemotherapies in adult patients with advanced gastrointestinal cancer. Among outcomes: effect on CSCs determined by immunohistochemistry NCT01775423 (phase I): in advanced malignancies NCT02231723 (phase I): with gemcitabine and nab-paclitaxel or chemotherapy combination in pancreatic cancer. Among outcomes: CSC assays on biopsied tumor tissue	(124)
Inhibitor of Nanog and other targets: BBI503 undisclosed structure	NCT02432690 (phase II): in asymptomatic recurrent ovarian cancer patients with CA-125 elevation NCT02232646 (phase II): in advanced urologic malignancies. Among outcomes: pharmacodynamics on biopsied patient tumor tissue NCT02232633 (phase II): in advanced hepatobiliary cancer. Among outcomes: pharmacodynamics on biopsied patient tumor tissue NCT02232620 (phase II): in advanced gastrointestinal stromal tumors. Among outcomes: pharmacodynamics on biopsied patient tumor tissue NCT02483247 (phase Ib/II): with selected anti-cancer therapeutics in adults with advanced cancer. Among outcomes: CSC assays on biopsied patient tumor tissue NCT01781455 (phase I): in advanced solid tumors. Among outcomes: effect on CSCs through immunogistochemistry NCT02432326 (phase Ib): BBI608 and BBI503 in advanced solid tumors NCT02279719 (phase Ib/II): BBI608 with sorafenib, or BBI503 with sorafenib in hepatocellular carcinoma. Among outcomes: CSC assays on biopsied patient tumor tissue.	(125)
AMPK activator metformin 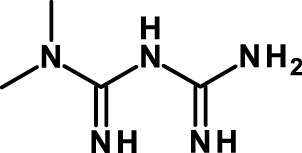	NCT01442870 (phase I): clinical safety of combining metformin with anti-cancer chemotherapy NCT01579812 (phase II): targeting CSCs for the prevention of relapse in stage IIC/III/IV ovarian, fallopian tube, and primary peritoneal cancer	(126) (127)
	NCT01717482 (phase II). Metformin for chemoprevention in NSCLC. Among outcomes: metformin sensitivity in induced pluripotent stem cells	(128–133)

*AMPK, 5′ adenosine monophosphate-activated protein kinase; CRC, colorectal cancer; CSC, cancer stem-like cell; FAK, focal adhesion kinase; mTOR, mammalian target of rapamycin; NSCLC, non-small cell lung cancer; PI3K, phosphoinositide 3-kinase; STAT, signal transducer and activator of transcription*.

Two FAK inhibitors that have been originated by the same company are now in clinical investigations for their antitumor and anti-CSC activity: defactinib/VS-6063/PF-04554878 and VS-4718. These are all small-molecule tyrosine kinase inhibitors. Results of a phase I study with defactinib have recently been reported (118). Disease stabilization at 12 weeks occurred in 6 of 37 patients receiving doses ≥200 mg/day. Treatment-related adverse events were mild to moderate, and reversible. A recommended phase II dose was defined. Of note, one clinical phase II study with defactinib in mesothelioma (NCT01870609) has been terminated due to lack of efficacy. Defactinib was also compared with a small-molecule FAK scaffolding inhibitor, Y15 (119). Cell viability was decreased in a dose-dependent manner in four thyroid cancer cell lines with Y15 and with higher doses of defactinib. Importantly, a combination of the two yielded synergistic effects, suggesting the possibility of enhancing efficacy by combining FAK inhibitors that work through different mechanisms of action. Defactinib is also being studied in a clinical trial in combination with VS-5584, a dual phosphoinositide 3-kinase (PI3K)-mammalian target of rapamycin (mTOR) inhibitor that will be discussed in the following. VS-4718 is in two clinical studies. In preclinical studies, VS-4718 showed that cells most sensitive to FAK inhibition lacked expression of the neurofibromatosis type 2 tumor suppressor gene product, Merlin (120). Merlin expression is often lost in malignant pleural mesothelioma. Low Merlin expression predicted for increased sensitivity of mesothelioma cells to VS-4718, *in vitro* and *in vivo*. Moreover, whereas pemetrexed and cisplatin, standard-of-care agents for mesothelioma, enrich for CSCs, FAK inhibitor treatment preferentially eliminated these cells.

PF-00562271 is another FAK inhibitor that was investigated in clinical studies, but its development has now been discontinued. In metastatic docetaxel-resistant prostate cancer cell lines, it reduced FAK phosphorylation in the resistant cells without affecting cell viability and overcame the chemoresistant phenotype (134).

#### Inhibitors of the PI3K–AKT–mTOR Pathway

The first component of this pathway, PI3K, encompasses three classes of molecules, with class IA PI3Ks being the most deeply investigated in cancer (135). Class IA PI3Ks are activated by RTKs, G-protein-coupled receptors, and some other post-receptor signaling molecules (e.g., RAS). Activated PI3K converts phosphatidylinositol 4,5-bisphosphate [PI(4,5)P_2_] into phosphatidylinositol 3,4,5-trisphosphate [PI(3,4,5)P_3_]. PI(3,4,5)P_3_ binds and recruits the second pathway component, AKT, to the plasma membrane. This process is negatively regulated by the tumor suppressor phosphatase and tensin homolog (PTEN), which converts PI(3,4,5)P_3_ back to PI(4,5)P_2_. AKT is activated at the plasma membrane through phosphorylation. Activated AKT initiates a cascade of downstream signaling events, which promote cellular growth, metabolism, proliferation, survival, migration, apoptosis, and angiogenesis. A major downstream effector of AKT is mTOR complex (mTORC) 1; its downstream targets control protein synthesis. Another mTORC, mTORC2, participates in the phosphorylation and activation of AKT (136). The PI3K–AKT–mTOR pathway is one of the most frequently activated signaling pathways in cancer. The two most common mechanisms in human cancers are activation by RTKs and somatic mutations in specific components of the signaling pathway (135).

The PI3K–AKT–mTOR pathway is involved in CSC biology in several solid tumors and hematological malignancies: leukemias (122, 137), breast cancer (138, 139), colon cancer (140), pancreatic cancer (85), SCLC (141), glioblastoma (142), and bladder cancer (138).

Given the relevance of this pathway in tumorigenesis, it is not surprising that the number of compounds that inhibit one or more components of these pathways and that are in clinical studies is enormous (135). Yet, few of them are being investigated as anti-CSC compounds. One is VS-5584, a PI3K/mTOR dual inhibitor. It exhibits approximately equal low nanomolar potency against mTOR and all PI3K class I isoforms (121). VS-5584 is up to 30-fold more potent in inhibiting the proliferation and survival of CSCs compared with non-CSCs in solid tumor cell populations. It preferentially diminishes CSC levels in mouse xenograft models and, *ex vivo*, in surgically resected breast and ovarian patient tumors. VS-5584 delayed tumor regrowth following chemotherapy in xenograft models of SCLC. The preferential activity on CSCs compared to non-CSCs may explain the limited efficacy of PI3K inhibitors used as monotherapies in trials on patients with tumors harboring PI3K pathway activation (143). VS-5584 is currently in phase I clinical trials in hematological and non-hematological malignancies, alone or in combination with the FAK inhibitor VS-6063 (Table 3).

BEZ235 is another PI3K/mTOR dual inhibitor that has prominent anti-CSC activity (144). Thus, combination of BEZ235 with radiotherapy effectively increased radiosensitivity of radioresistant prostate cancer cell lines, induced more apoptosis in radioresistant cells, reduced the expression of EMT and CSC markers and of the PI3K–AKT–mTOR pathway compared with radiotherapy alone. The PI3K–AKT–mTOR signaling pathway is highly activated also in colon CSCs and inhibition with BEZ235 suppressed their proliferation (140). BEZ235 has been investigated in numerous clinical studies for its antitumor activity, but the development has now been discontinued.

Eventually, also the mTOR inhibitor temsirolimus, which has already been approved for the therapy of renal cell carcinoma is being evaluated in a phase I/II clinical trial in combination with liposomal doxorubicin in patients with advanced soft tissue and bone sarcomas. Clinical efficacy and proportion of CSCs before and after therapy are some of the outcomes of this study (Table 3).

#### Inhibitors of the RAS–RAF–MEK–ERK Pathway

The RAS–RAF–MEK-extracellular signal-regulated kinase (ERK) signaling pathway is, together with the PI3K–AKT–mTOR pathway, one of the most frequently involved in cancer biology. In fact, this pathway is hyperactivated in a high percentage of tumors, mostly because of the presence of activating mutations of the *RAS* genes (145). Recently, several compounds targeting components of this pathway have been approved for the therapy of metastatic melanoma and have shown promising clinical activity in other tumor types (68, 146). Several preclinical studies showed an involvement of this signaling pathway in the CSC biology of several tumor types. Thus, deficient expression of a negative regulator, dual specificity phosphatase-4, leads to aberrant activation of the pathway with consequent resistance to chemotherapeutics, increased mammosphere formation of CD44^+^/CD24^−^ tumor cells in basal-like breast cancer (147). This pathway is also involved in the biology of CSCs of several tumor types, such as bladder cancer (148), colon cancer (149), and leiomyosarcoma (150). In some cases, a coordinate activation of the PI3K–AKT–mTOR and RAS–RAF–MEK–ERK has been demonstrated (150, 151). This finding is not surprising, given the intimate relationship between these two pathways, with RAS being an activating signaling node for both pathways (152).

A MEK inhibitor, trametinib (GSK1120212), is being tested in a phase II clinical trial in surgically resectable oral cavity squamous cell cancer (Table 3). Measurement of changes of CD44^+^ CSCs before and after treatment is one of the outcomes of this study. In fact, a preclinical study in a model of head and neck cancer had shown that CD44 is a critical target of ERK in promoting tumor aggressiveness and proposed this pathway as a target to treat head and neck cancer (123).

#### Inhibitors of Signal Transducer and Activator of Transcription 3 and Other Transcription Factors

Signal transducer and activator of transcription (STAT) 3 is a transcription factor downstream of several cytokine (e.g., IL-6) and growth factor receptors and non-RTKs, such as SRC (153, 154). STAT3 is frequently overexpressed in carcinomas secondary to the activation of upstream kinases (155). It modulates the expression of a broad range of downstream genes (156), and plays a crucial role in CSC biology in several tumor types, such as breast cancer (139), colon cancer (157), endometrial cancer (158), prostate cancer (159), lung cancer (160), pancreatic cancer (161), and glioblastoma (162).

Napabucasin/BBI608 is a synthetic napthofurandione natural product, which was originally extracted from *Bignoniaceae tabeluia*. It inhibits gene transcription driven by STAT3, blocks spherogenesis of CSCs, kills CSCs, and downregulates CSC-associated genes (124). *In vivo*, BBI608 prevents cancer relapse in a pancreatic cancer model and metastasis but completely spares hematopoietic stem cells. BBI608 is well tolerated with no signs of adverse events in preclinical toxicology studies. BBI608 is in phase III clinical trials (Table 3). While BBI608 is the most advanced anti-CSC compound in clinical development, there are concerns about the specificity of this compound, given its quinone structure and potential to exert pleiotropic effects inside cells. Risks and opportunities of such promiscuous compounds have been recently discussed (163, 164).

The same company is developing also another small-molecule inhibitor, BBI503. While no publication is available about this inhibitor, it is claimed to inhibit Nanog and other CSC pathways by targeting kinases. Nanog is a transcription factor that acts as a key regulator of embryonic stem cell maintenance. Nanog is dysregulated in cancer and is involved in the maintenance of CSCs (125). BBI503 is in several phase I–II clinical studies, and two clinical studies are also investigating the combined use of BBI608 and BBI503 (Table 3).

#### Activators of 5′ Adenosine Monophosphate-Activated Protein Kinase

While most anti-CSC compounds are inhibitors of pathways that are conducive to CSCs, 5′ adenosine monophosphate-activated protein kinase (AMPK) acts as an endogenous inhibitor of CSCs and, consequently, activators of AMPK are being investigated as anti-CSC compounds. The anti-diabetic drug metformin is the prototypic member of this class. It inhibits preferentially the growth of CSCs compared to the bulk of tumor cells and combinatorial therapy with standard chemotherapeutics (doxorubicin, paclitaxel, and cisplatin) increases tumor regression and prolongs remission in mouse xenografts (126). In addition, metformin can decrease the chemotherapeutic dose for prolonging tumor remission in xenografts of multiple cancer types. Phenformin, a related biguanide, is generally considered to represent a stronger version of metformin. While the most broadly accepted mechanism of action whereby metformin inhibits CSCs is through AMPK activation and consequent inhibition of PI3K–AKT–mTOR signaling (127), other AMPK-independent mechanisms may contribute to the antitumor activity of metformin (128, 165). Metformin has also been shown to inhibit and reverse EMT in cell lines resistant to EGFR tyrosine kinase inhibitors through inhibition of TGF-β-induced EMT (131) and of the IL-6/STAT3 axis (132). Recent results in a non-tumor system have shown that metformin inhibits monocyte-to-macrophage differentiation via AMPK-mediated inhibition of STAT3 activation (133). These observations establish a link between AMPK and another crucial pathway in CSC biology, STAT3, in addition to PI3K–AKT–mTOR. Metformin is now being studied in a large number of clinical trials for its antitumor activity, and some of these trials investigate also the anti-CSC activity (Table 3).

### Inducers of CSC Differentiation

The possibility of inducing the differentiation of CSCs into non-CSC tumor cells appears a logical approach for anti-CSC therapy. It is speculated that some of the anti-CSC compounds discussed so far act by inducing differentiation of CSCs (98). In the following, we discuss some other compounds that act by inducing CSC differentiation.

All-trans-retinoic acid/tretinoin is currently used for the therapy of acute promyelocytic leukemia (APL), a subtype of AML, where it induces differentiation of leukemic blasts. However, among patients with non-APL AML, tretinoin-based treatment has not been effective. It has now been shown that, through epigenetic reprograming, inhibitors of lysine-specific demethylase 1, including tranylcypromine, unlock the tretinoin-driven therapeutic response in non-APL AML (166). Treatment with tretinoin plus tranylcypromine markedly diminished the engraftment of primary human AML cells *in vivo* in immunodeficient mice, suggesting that tretinoin in combination with tranylcypromine may target leukemia CSCs. This therapeutic combination is now being studied in a phase I clinical trial in AML patients (Table 4).

**Table 4:** **Inducers of CSC differentiation**.

Anti-CSC compound	ClinicalTrials.gov Identifier (phase for the indicated trials): Patient population, CSC-related outcomes	Reference
Tretinoin 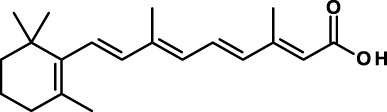	NCT02273102 (phase I): with tranylcypromine in acute myeloid leukemia and myelodysplastic syndromes	(166)
Arsenic trioxide 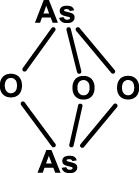	NCT01397734 (phase I): targeting leukemia stem cells with arsenic trioxide and TKIs in chronic myeloid leukemia	(167)
Thioridazine 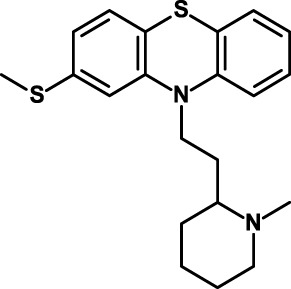	NCT02096289 (phase I): with intermediate-dose cytarabine in older patients with relapsed or refractory acute myeloid leukemia. Among outcomes: assessment of functional leukemia stem cells	(168)

*CSC, cancer stem-like cell; TKI, tyrosine kinase inhibitor*.

Also arsenic trioxide is used for the therapy of APL where it induces differentiation and apoptosis of leukemic cells. Arsenic trioxide has now been reported to induce differentiation of HCC CSCs by downregulating the expression of CD133 and other CSC markers (167). The self-renewal ability and tumorigenic capacity, but not the proliferation *in vitro*, were inhibited. *In vivo*, arsenic trioxide decreased recurrence rates after radical resection and prolonged survival in a mouse model. Arsenic trioxide is being studied, in combination with tyrosine kinase inhibitors in a clinical trial for its capacity to target the CML stem cell population (Table 4).

Thioridazine is an antipsychotic drug that antagonizes dopamine receptors. It was identified using a discovery platform that reveals differences between neoplastic and normal human stem cells in undergoing differentiation in response to molecules from libraries of known compounds (168). Thioridazine was found to selectively target neoplastic cells, and to impair human somatic CSCs capable of *in vivo* leukemic disease initiation while having no effect on normal blood stem cells. Thioridazione is now undergoing a phase I trial in combination with cytarabine in patients with AML. Objective response rates and effects on leukemic stem cells are among the outcomes of this study (see Table 4).

### Radiotherapeutic Targeting of CSC-Enriched Domains

CSCs are resistant to radiation because of enhanced self-renewal capacity, DNA-repair capacity, and enhanced reactive oxygen species defenses. In spite of this radioresistance, new radiotherapeutic approaches appear to be promising treatments for the targeting of CSCs (169). Moreover, it is currently believed that glioblastoma CSCs derive from the transformation of normal tissue stem cells residing in the subventricular zones of the brain (170). This finite anatomic location of the glioblastoma CSC pool has suggested the possibility of targeting these cells with radiotherapeutic approaches. One clinical trial investigating the effects of radiotherapy to brain areas containing CSCs in combination with temozolomide in glioblastoma patients is ongoing (NCT02039778, phase I/II).

### Anti-CSC Compounds with Undefined Mechanism(s) of Action or Targeting Diverse Signaling Pathways

#### Salinomycin

Salinomycin is the first molecule that was found to have anti-CSC activities. It was identified in a chemical screen that had been designed to discover compounds showing selective toxicity for breast CSCs (171). Salinomycin has been widely used as an anticoccidiosis agent in chickens. It is a natural, fused polypyran ionophore able to shield charge of ions and to cross cell lipid membranes, thereby interfering with transmembrane potassium potential and promoting mitochondrial and cellular potassium flux. Salinomycin is a complex natural molecule produced by fermentation with many stereocenters. Efforts are underway to develop synthetic salinomycin analogs. Salinomycin reduced the proportion of CSCs by >100-fold relative to paclitaxel, inhibited mammary tumor growth *in vivo* and reduced expression of breast CSC genes. Ionic changes induced by salinomycin were found to inhibit proximal WNT signaling by interfering with phosphorylation of the WNT coreceptor LPR6 (172). In the following, salinomycin has also been found to modulate Hh signaling (173), to induce accumulation of reactive oxygen species (174), and inhibit STAT3 activation (175). In general, compounds endowed with a pleiotropic array of mechanisms of action are, clinically, of little promise. Nevertheless, salinomycin has been reported being investigated in clinical trials (176), but these trials are not reported under the clinicaltrials.gov website.

#### Disulfiram

This is a dithiocarbamate used for the treatment of chronic alcoholism where it produces acute sensitivity to ethanol due to its inhibitory activity on ALDH (177). Disulfiram was found to possess antitumor activity in early clinical studies (178) and, in recent years, to possess anti-CSC activities. Thus, disulfiram inhibits TGF-β-induced EMT and CSC-like features in breast cancer cells (179). It can also inhibit the proliferation and self-renewal of glioblastoma CSCs (180). Regarding the mechanism of action, disulfiram can act as a proteasome inhibitor and this, in turn, inhibits nuclear translocation and DNA-binding activity of NF-κB (130). Moreover, being an irreversible inhibitor of ALDH, disulfiram might also act as an inhibitor of ALDH-positive CSCs (129). Disulfiram is now being investigated in clinical studies, and the results of a preliminary clinical trial appear encouraging (181). In one study, it is used in combination with temozolomide in newly diagnosed glioblastoma (Table 5). This study refers explicitly to the anti-CSC activity of disulfiram.

**Table 5:** **Anti-CSC compounds with undefined mechanism of action or targeting diverse signaling pathways**.

Anti-CSC compound	ClinicalTrials.gov Identifier (phase for the indicated trials): patient population, CSC-related outcomes	Reference
Disulfiram 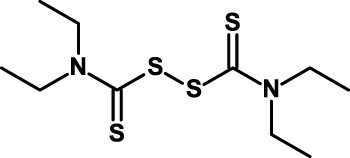	NCT01777919 (phase II): disulfiram and copper with chemotherapy in the treatment of newly diagnosed glioblastoma	(59, 129, 130, 165, 180, 181)
Chloroquine 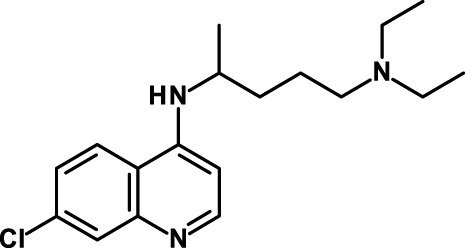	NCT01023477 (phase I/II): testing whether chloroquine reduces the ability of ductal carcinoma *in situ* to survive and spread. Among outcomes: effect on progenitor cell yield and invasive capacity *ex vivo*	(182, 183)
Oncolytic adenovirus	NCT01956734 (phase I): with temozolomide for treatment of glioblastoma – combination effective in killing glioblastoma CSCs	(184)

*CSC, cancer stem-like cell*.

#### Chloroquine

Chloroquine has anti-CSC activity (182), and this effect is considered to be the consequence of the lysosomotropic action of chloroquine and consequent inhibition of autophagy. Balic et al. (183), however, have found that chloroquine targets pancreatic CSCs through a dual mechanism of action: first, inhibition of CXC ligand 12/CXCR4 signaling, resulting in reduced phosphorylation of ERK and STAT3. Second, chloroquine inhibited Hh signaling by decreasing the production of SMO, translating into downregulation of downstream targets in CSCs and the surrounding stroma. Chloroquine-induced inhibition of STAT3 signaling as a mechanism for the anti-CSC activity of chloroquine has been reported also by Choi et al. (185). Chloroquine is currently being investigated in clinical studies for its anti-CSC activity (see Table 5).

#### Oncolytic Adenovirus

The tumor-selective oncolytic adenovirus Delta-24-RGD has antiglioma effects (186) and overcomes temozolomide resistance by silencing the O^6^-methylguanine-DNA methyltransferase promoter (187). Delta-24-RGD infects, replicates, and induces autophagic cell death in brain CSCs (184). It is being studied in combination with temozolomide in a phase I clinical trial in glioblastoma patients (Table 5).

### Vaccination Against CSCs

Vaccination against CSCs or individual proteins overexpressed in CSCs is another anti-CSC approach. In preclinical studies, syngeneic hosts have been immunized with CSC-enriched populations (188). This source was more effective than unselected tumor cells in inducing protective antitumor immunity. Immune sera from vaccinated hosts contained lytic anti-CSC IgG antibodies, and cytotoxic T-lymphocytes that were capable of killing CSCs *in vitro*.

Recently, anti-CSC vaccination against individual proteins has been proposed. Sox2 is a transcription factor required for the maintenance of normal neural stem cells and for the biology of oligodendroglioma CSCs. Immunization of mice with Sox2 peptides delayed tumor development and prolonged survival (189). In another study, xCT, the functional subunit of the cystine/glutamate antiporter system xc-, has been found to be upregulated in mammospheres derived from murine human EGFR2-positive breast tumor cells (190). Downregulation of xCT impaired mammosphere generation and altered CSC intracellular redox balance *in vitro*. DNA vaccination-based immunotargeting of xCT in mice delayed subcutaneous growth of mammophere-derived cells, impaired pulmonary metastasis formation, and increased CSC chemosensitivity to doxorubicin *in vivo*. Table 6 lists ongoing clinical trials aimed at vaccinating tumor-bearing patients against CSCs.

**Table 6:** **Anti-CSC vaccines**.

Molecular target, anti-CSC compound	ClinicalTrials.gov Identifier (phase): clinical trial indication	Reference
CSC-loaded DCs	NCT02178670, NCT02074046, NCT02115958, NCT02084823, NCT02089919, NCT02176746, NCT02063893 (phase I/II): vaccination against ovarian cancer, pancreatic cancer, nasopharyngeal cancer, lung cancer, HCC, CRC, breast cancer	(188)
Multiantigen DNA plasmid-based vaccine	NCT02157051 (phase I): in HER2-negative advanced stage breast cancer	
Stem cell, tumor-amplified RNA loaded on DCs	NCT00890032 (phase I): in patients undergoing surgery for recurrent glioblastoma multiforme	
DCs transfected with CSC-derived mRNA	NCT00846456 (phase I/II): in patients receiving standard therapy for glioblastoma	(191)
Autologous DCs pulsed with lysate from an allogeneic glioblastoma CSC line	NCT02010606 (phase I): in newly diagnosed or recurrent glioblastoma. Among outcomes: assessment of CSC antigen expression	
DCs loaded with glioma CSCs-associated antigens	NCT01567202 (phase II): in glioblastoma multiforme.	(192)
DCs loaded with brain tumor CSCs	NCT01171469 (phase I): in recurrent or progressive malignant gliomas	
Peptides WT1: 126–134 and PRI:169–177, found in leukemic stem cells, in MontanideTM and administered with GM-CSF	NCT00488592 (phase II): in low risk myeloid malignancies	(193)

*CRC, colorectal cancer; CSC, cancerstem-like cell; DC, dendritic cell; GM-CSF, garnulocyte-macrophage colony-stimulating factor; HER, human epidermal growth factor; HCC, hepatocellular carcinoma*.

### Looking Ahead – 1. Epigenetic Drugs for Anti-CSC Therapy

Epigenetic reprograming is a characteristic trait of tumorigenesis and CSC biology. Reprograming has been demonstrated for every component of the epigenetic machinery, including DNA methylation, histone modifications, nucleosome positioning, and non-coding RNAs, specifically microRNA expression (194). The possibility of therapeutically manipulating these events is attractive because cancer-associated epigenetic aberrations are reversible. Not surprisingly, drug discovery in epigenetics is being widely pursued and four epigenetic drugs have already received FDA approval (azacitidine, decitabine, vorinostat, and romidepsin). Inhibition of CSCs with epigenetic drugs has been demonstrated in several instances (195–197), and this is likely to become a rapidly progressing domain. A note of caution to be sounded for these drugs comes from the knowledge that some epigenetic drugs have been reported to have tumor-promoting or tumor-inhibiting activities in a context-dependent manner (31, 194).

### Looking Ahead – 2. Improving Tumor Delivery of Anti-CSC Medicines

CSCs are often reported to be concealed in hypoxic tumor areas (198), and these areas pose significant problems for drug penetration (199). Therefore, administration of anti-CSC compounds may not achieve complete depletion of CSCs present at any given time, because of inadequate tumor penetration. Moreover, inadequate penetration of antitumor drugs may contribute to the induction of drug resistance (200). Therefore, improving the delivery of anti-CSC compounds to their target cells has become an area of active research (201). In general, one can envisage two main approaches. First, through the use of passively or actively targeted nanoparticles (202). Second, through coadministration of promoter drugs that facilitate the penetration of compounds endowed with direct antitumor effects (the effector drug) (199, 203). While none of these approaches has yet made its way to the clinic, several preclinical studies suggest their feasibility. Thus, PEGylated polymeric micelles were loaded with salinomycin to effectively target breast CSCs and shown to be more effective than salinomycin *in vivo* (204). Others demonstrated that nanoparticles can penetrate, upon convection-enhanced delivery, large intracranial volumes in experimental animals. When loaded into these nanoparticles, a compound that had been identified possessing anti-CSC activity, ditiazanine iodide, significantly increased survival in rats bearing brain CSC-derived xenografts (205).

Regarding the possibility of improving the penetration of effector drugs through the coadministration of a promoter drug, it is interesting to note that an anti-CSC compound that has been discussed above, IPI-926, a Hh pathway inhibitor, has been shown to improve penetration of an effector drug (gemcitabine) into pancreatic tumors through depletion of tumor-associated stromal tissue (206). In fact, paracrine Hh signaling from neoplastic cells promotes stromal desmoplasia (207). Combined with their anti-CSC activity, compounds of this class may be of particular interest because they have the potential to combine in a single molecule anti-CSC activity and promotion of tumor penetration.

## Anti-CSC Compounds in Clinical Development – A Critical Appraisal

There are several crucial points to address when taking an overall look to anti-CSC compounds in current clinical investigation.

The first, most important and most difficult point to answer is how to evidence clinical benefit that may derive from anti-CSC compounds. Currently used antitumor drugs target mainly proliferating tumor cells, while sparing CSCs and even inducing the generation of new CSCs. On the other hand, anti-CSC compounds are inactive against the bulk of proliferating tumor cells because they target markers or pathways that are overexpressed or selectively expressed on CSCs compared to bulk tumor cells. Consequently, the vast majority of ongoing clinical trials with anti-CSC compounds are performed in combination with other antitumor drugs belonging to different classes of compounds. Therefore, the easiest way to answer the question as to how evidence clinical benefit it to say that anti-CSC compounds should improve the efficacy of anti-cancer therapies that are given in combination, i.e., higher percentages of patients with clinical responses or stable disease for longer periods of time than patients treated with standard-of-care therapies. This, however, is the goal of any anti-cancer therapy, whether or not targeting CSCs. So, the question arises as to how it is possible to recognize *bona fide* anti-CSC activity compared to generic antitumor effects.

A first possibility, and probably also the most realistic, is to state that any clinical benefit deriving from anti-CSC therapy should be accompanied by a decline of tumor tissue CSCs or circulating CSCs (208). Not surprisingly, many clinical trials with anti-CSC compounds include, among the study outcomes, the determination of tumor tissue or circulating CSCs (see the tables throughout this article). In fact, it is not obvious that anti-CSC compounds exert antitumor effects through their anti-CSC activity, because most, if not all of them, are not CSC-specific. One example is represented by anti-DLL4 antibodies. While one anti-DLL4 antibody is being developed as an anti-CSC compound (79), a second antibody is being developed as an anti-angiogenic compound (108), and anti-DLL4 antibodies have even been shown to exert antitumor effects by acting on cells of the tumor stroma (107).

In spite of such a pharmacodynamic approach it would be, nevertheless, desirable to identify the clinical setting that would profit most from anti-CSC therapy. If we consider that CSCs replenish the bulk of proliferating tumor cells, then anti-CSC compounds should be most efficacious after debulking of tumor cells, i.e., in an adjuvant setting. In fact, in tumors that do not display HER2 gene amplification, HER2 may be selectively expressed on CSCs, and an anti-HER2 antibody blocked growth of these HER2-negative tumors in mouse models when administered in the adjuvant setting but had no effect on established tumors (209). Given that, it is somehow surprising to note that there are no ongoing clinical trials studying anti-CSC compounds in an adjuvant setting. Only one study evaluates the expression of a CSC marker as a predictor of adjuvant chemotherapy response in breast cancers of high-risk women (NCT00949013). A likely reason for this attitude is that the execution of clinical trials in an adjuvant setting is challenging for most tumor types, both in terms of duration and costs.

Another question is as to which molecular targets should be addressed in order to have the greatest chances of success. Although, also in this case, only results from clinical practice will give definitive answers, some guidelines may help in taking the most promising roads. First, going through the pathways that are involved in CSC biology and are targets for anti-CSC compounds, it can be appreciated that none of these pathways or targets have been clearly attributed to a single tumor type or subtype. This implies that in each tumor several of these pathways can be active, i.e., the system appears highly redundant and prone to native and acquired resistance. There are several possibilities to mitigate these consequences.

First, using a given anti-CSC compound only when aberrant activation of the relevant pathway is clearly demonstrated. This is important not only for avoiding native drug resistance and delaying insurgence of acquired drug resistance, but also because some of the pathways that are being targeted for anti-CSC therapy play a context-dependent tumor- and CSC-promoting role, while, in a different context, they may also exert tumor-suppressive effects. There are several examples of these opposite, context-dependent effects, for example, the Notch (210, 211), TGF-β (212), WNT (82, 213), and STAT3 (214) pathways.

Second, using anti-CSC compounds that are directly cytotoxic, such as ADCs or native antibodies that act through antibody-dependent cellular cytotoxicity or complement-dependent cytotoxicity. Use of these compounds does not exclude the possibility of selecting for cells that are mutated in the epitope recognized by the targeting moiety, as has been elegantly demonstrated in a recent work (215). Nevertheless, these compounds are expected to be less conducive to resistance than anti-CSC compounds that inhibit signaling pathways without being directly cytotoxic. In fact, these latter compounds may themselves promote acquisition of drug resistance when acting on cells at suboptimal concentrations like those that are achieved in hypoperfused tumor domains (200).

A further turn of complexity is given by the fact that, in any given tumor and at any given time, CSCs may represent a heterogeneous population, both functionally and phenotypically (116, 216). This fact, taken together with the redundancy of the signaling systems underlying CSC biology, suggests that the combined use of anti-CSC compounds may represent still another approach to mitigate untoward consequences. Thus, combined use of different anti-CSC compounds may embrace a larger part of the whole CSC population than a single compound. Preclinical studies suggest that, indeed, combined use of anti-CSC compounds is more efficacious than their individual use (85, 217), and, as already mentioned before in this article, clinical studies with combinations of anti-CSC compounds are ongoing.

Another last point to consider is the plasticity of CSCs. We have already discussed the role of stimuli from the tumor micronevironment in CSC biology. These stimuli are the result of unlimited tumor cell proliferation that causes the generation of different forms of stressors, which, in combination with the inherent genetic instability of tumor cells, shapes the CSC landscape. The implicit consequence of this is that once tumor cells resume growth after drug-induced depletion, the same conditions that generated the CSC-inducing stimuli will be recreated and give rise again to a CSC population. Therefore, anti-CSC therapy should not be expected to act as a device that leads, in combination with other antitumor therapies, to tumor eradication but, rather, as a tool to improve the clinical efficacy of existing drugs. The ongoing clinical trials will tell us in the next years if this is a feasible goal to achieve.

## Author Contributions

FM, CR, and FL contributed to the conception of the work, drafted or revisited it critically, approved the final version, and agreed to be accountable for all aspects of the work.

## Conflict of Interest Statement

The authors declare that the conception and preparation of this article was conducted in the absence of any commercial or financial relationships that could be construed as a potential conflict of interest.
